# Scaling Up Misoprostol to Prevent Postpartum Hemorrhage at Home Births in Mozambique: A Case Study Applying the ExpandNet/WHO Framework

**DOI:** 10.9745/GHSP-D-18-00475

**Published:** 2019-03-22

**Authors:** Karen Hobday, Jennifer Hulme, Ndola Prata, Páscoa Zualo Wate, Suzanne Belton, Caroline Homer

**Affiliations:** a Menzies School of Health Research, Charles Darwin University, Casuarina, Australia.; b Department of Family and Community Medicine, University of Toronto, Toronto, Canada.; c Bixby Center for Population, Health and Sustainability, University of California–Berkeley, Berkeley, CA, USA.; d Department of Women's and Child Health, Ministry of Health, Maputo, Mozambique.; e Burnet Institute, Melbourne, Australia.

## Abstract

**Background:**

Mozambique has a high maternal mortality ratio, and postpartum hemorrhage (PPH) is a leading cause of maternal deaths. In 2015, the Mozambican Ministry of Health (MOH) commenced a program to distribute misoprostol at the community level in selected districts as a strategy to reduce PPH. This case study uses the ExpandNet/World Health Organization (WHO) scale-up framework to examine the planning, management, and outcomes of the early expansion phase of the scale-up of misoprostol for the prevention of PPH in 2 provinces in Mozambique.

**Methods:**

Qualitative semistructured interviews were conducted between February and October 2017 in 5 participating districts in 2 provinces. Participants included program stakeholders, health staff, community health workers (CHWs), and traditional birth attendants (TBAs). Interviews were analyzed using the ExpandNet/WHO framework alongside national policy and planning documents and notes from a 2017 national Ministry of Health maternal, newborn, and child health workshop. Outcomes were estimated using misoprostol coverage and access in 2017 for both provinces.

**Results:**

The study revealed a number of barriers and facilitators to scale-up. Facilitators included a supportive political and legal environment; a clear, credible, and relevant innovation; early expansion into some Ministry of Health systems and a strong network of CHWs and TBAs. Barriers included a reduction in reach due to a shift from universal distribution to application of eligibility criteria; fear of misdirecting misoprostol for abortion or labor induction; limited communication and understanding of the national PPH prevention strategy; inadequate monitoring and evaluation; challenges with logistics systems; and the inability to engage remote TBAs. Lower coverage was found in Inhambane province than Nampula province, possibly due to NGO support and political champions.

**Conclusion:**

This study identified the need for a formal review of the misoprostol program to identify adaptations and to develop a systematic scale-up strategy to guide national scale-up.

## Abstract (precis)

Facilitating factors for this community-level scale up in 35 districts included strong government support, local champions, and a national policy on preventing postpartum hemorrhage (PPH). Challenges included a lack of a systematic scale-up strategy, limited communication of the PPH policy, a shift from a universal distribution policy to application of eligibility criteria, difficulties engaging remote traditional birth attendants, and implementation of a parallel M&E system.

## INTRODUCTION

Each day around the world, approximately 830 women die giving birth or due to complications during pregnancy, childbirth, and the following weeks.^1^ Urgent investment in maternal, newborn, and child health (MNCH) programming is needed in order for countries to achieve Sustainable Development Goal (SDG) 3.1—reduce global maternal mortality to less than 70 per 100,000 live births by 2030.^2^ Scale up of high-impact MNCH interventions that reduce maternal mortality and benefit entire populations is a growing public health and political priority.^3^^–^^6^

Postpartum hemorrhage (PPH) remains one of the leading causes of maternal mortality globally and the number one cause in sub-Saharan Africa.^7^ Mozambique, located in Southern Africa, has a high maternal mortality ratio (MMR) of 489 per 100,000 live births.^8^ PPH is one of the main causes of maternal deaths in Mozambique; estimates range from 30.7%^9^ to 38.0% of maternal deaths.^10^ The Ministry of Health (MOH) has prioritized the prevention of maternal mortality in its *Health Sector Strategic Plan 2014–2019*.^11^

Postpartum hemorrhage is one of the main causes of maternal deaths in Mozambique.

Access to rural health services in Mozambique is limited by distance, lack of transportation, health facility coverage, and quality.^12^^,^^13^ In 2013 there were 0.05 physicians and 0.40 nurses and midwives per 1,000 population.^14^ This is significantly below the WHO standard for health worker density of 2.5 doctors, midwives, and nurses per 1,000 population.^15^ With inadequate resources to train and retain health staff,^16^ Mozambique experiences both brain drain and internal migration.^17^

Nationally, an estimated 30% of Mozambican women give birth at home without assistance from a skilled birth attendant.^18^ Women who give birth at home often have support from a traditional birth attendant (TBA) or family member/friend. In Inhambane province, a reported 89.1% of women gave birth with a skilled birth attendant, 3.9% with a TBA, 6.6% with family/friends, and 0.4% alone in the 2 years preceding the 2015 Demographic and Health Survey. In Nampula province, 74.4% of women gave birth with a skilled birth attendant, 11.7% with a TBA, 13.2% with family/friends, and 0.7% alone.^18^

Oxytocin, an injectable uterotonic, is the first-line therapy to prevent and treat PPH and is available in the majority of health facilities across Mozambique.^19^ However, oxytocin must be administered by a trained health worker as an injection and ideally stored in a refrigerator.^20^ In many low-income contexts, misoprostol, a heat-stable tablet that can be used as an alternative uterotonic, can be administered orally by a community health worker (CHW) or the woman herself.^21^ However, opponents of misoprostol for the prevention of PPH often fear that it will be used incorrectly or for abortion.

In 2014, the abortion laws in Mozambique were changed to permit women to have a safe abortion up to 12 weeks, up to 16 weeks in cases of rape or incest, and up to 24 weeks if there are fetal abnormalities.^22^ As of 2018, women can access abortion in primary, secondary, and tertiary health facilities that have received training and resources to carry out the procedure.^23^

In 2009–2010, Bique et al. studied the use of misoprostol for the prevention of PPH in home births in Mozambique.^24^ The pilot study was conducted in response to the MOH request for local research to establish the safety of using misoprostol in the community. Misoprostol was distributed in advance to pregnant women via MNCH staff during antenatal care visits and through direct administration to women from TBAs. Results revealed that TBAs and women themselves could safely and effectively administer misoprostol at home births. In 2011, the results of the pilot were presented to the Mozambique MOH, which subsequently approved the scale-up of the use of misoprostol for the prevention of PPH. For more information about the role of TBAs and CHWs in Mozambique, see Box 1.

BOX 1 **The Role of TBAs and CHWs in Mozambique**

TBAs play a supportive role for pregnant women, providing assistance during birth and the postpartum period. In Mozambique, TBAs are female and generally illiterate. They do not receive a salary, and they work with very few resources. For example, they do not receive clean birthing kits. The majority of Mozambican TBAs do not have formal training or certification in child birth practices or obstetric emergencies.^25^^,^^26^

The TBAs involved in the misoprostol program are affiliated with the participating health facilities. They receive a 3-day training on how to safely administer misoprostol to women after they give birth, distribute chlorhexidine, and recognize danger signs for referral and safe birth practices. The training emphasizes the role of TBAs in referring or accompanying women to the health facility to give birth. When a TBA is aware of an impending birth in the community, she requests a dose of misoprostol from the CHW in her catchment area. The TBA is responsible for safeguarding that dose until she attends the birth and administers it directly to the woman after she gives birth. TBAs and CHWs do not distribute misoprostol to pregnant women.

CHWs in Mozambique are referred to as *Agentes Polivelantes Elementares* (APEs) in Portuguese. The vast majority (85%) of APEs are men, mainly due to the prerequisite of primary school education and the need to undertake a 4- to 5-month training, often outside their community. The APE program is now actively working to improve gender equality by recruiting more women.^27^ APEs receive a monthly salary of 1,200 meticals (approximately US$18 in 2018) and are expected to cover a catchment area of 8 to 25 km from the health facility with which they are affiliated.

Integrated community case management of malaria, pneumonia, and diarrhea is a key component of the work that CHWs undertake. The CHWs receive a small medical kit and have the authority to provide medicines, including antimalarials, amoxicillin, and zinc.^28^ In 2016, 4 new products were added to their role: family planning including administration of injectable contraceptives, vitamin A, chlorhexidine, and distribution of misoprostol to TBAs.

**FIGURE 1:**
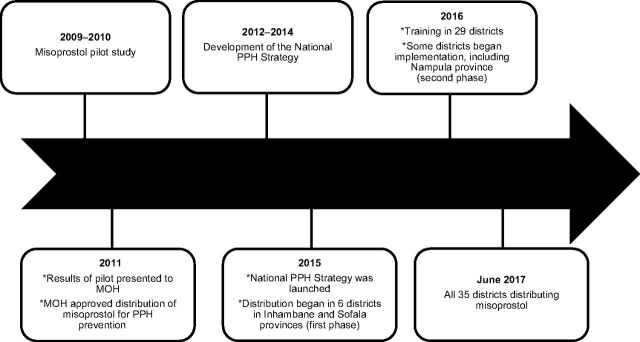
The Evolution of the Distribution of Misoprostol for Prevention of Postpartum Hemorrhage in Mozambique

In 2015, the MOH launched the national *Strategy for the Prevention of Postpartum Hemorrhage at the Community Level* (referred to as the National PPH Strategy). The first objective of the strategy was the implementation of the misoprostol program, which included advance distribution of misoprostol to women during pregnancy and direct administration by TBAs to women who give birth in the community to reduce maternal mortality associated with PPH.^29^ The National PPH Strategy included a 2-year general plan of activities with the intention that each province would develop a specific action plan for program implementation. The target was to roll out the misoprostol program in 35 districts in 10 provinces. The MOH opted for a stepwise approach, initially limiting implementation to 6 districts in 2 provinces—3 in Inhambane and 3 in Sofala. The intent was to learn from the experience of the first 6 districts before further expansion. The second phase of expansion took place in 2016–2017 in 29 districts across 8 provinces. By June 2017, the misoprostol program was being implemented in 35 districts. See Figure 1 for a timeline of events.

**FIGURE 2:**
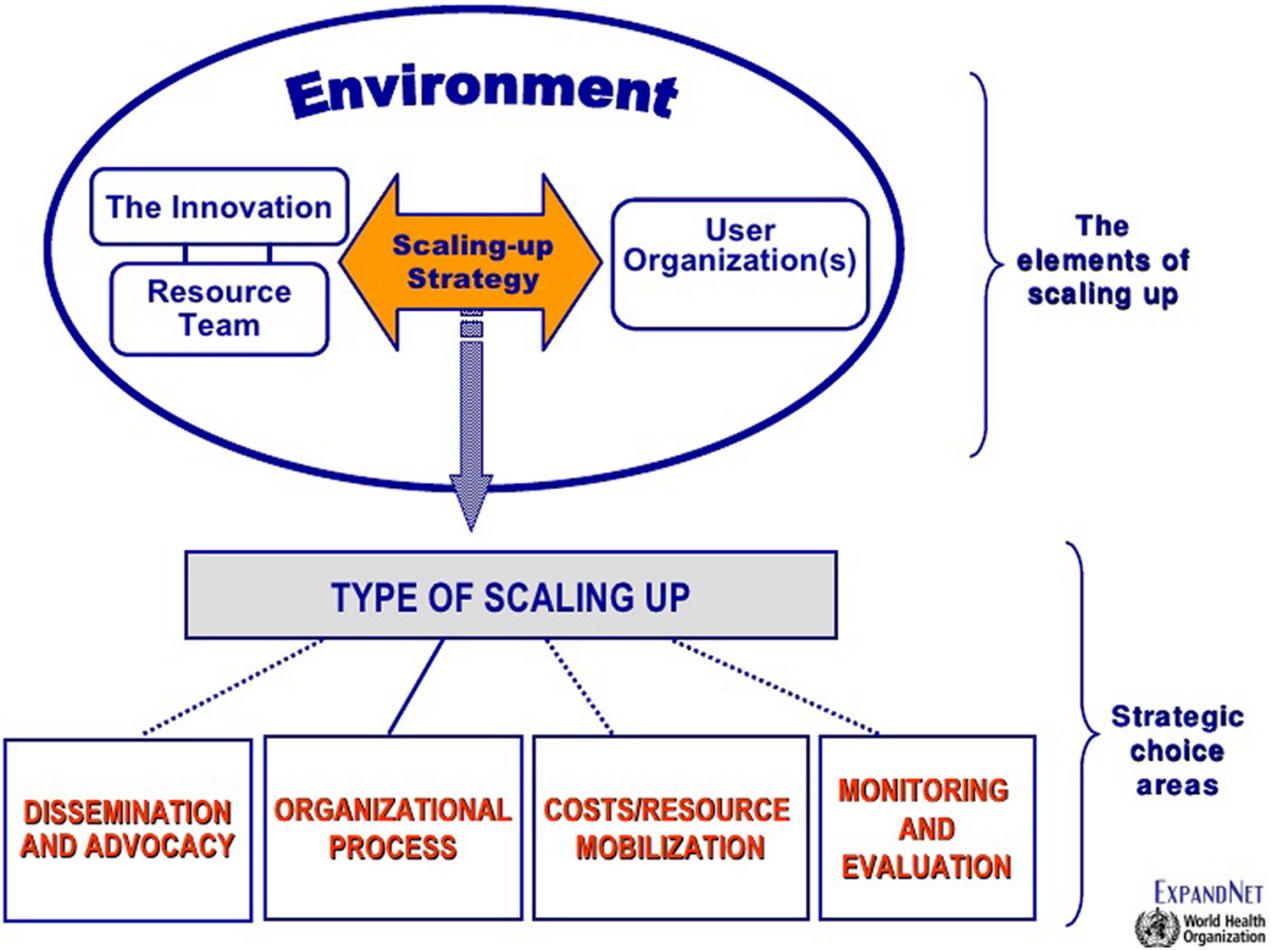
ExpandNet/World Health Organization Scale-Up Framework

There is an urgent need for information about the process of scale-up of MNCH interventions that highlights the operational realities countries experience.^30^^,^^31^ ExpandNet, in collaboration with the World Health Organization (WHO), defines scale-up, as the “… deliberate efforts to increase the impact of health service innovations successfully tested in pilot or experimental projects so as to benefit more people and to foster policy and programme development on a lasting basis.”^32^ We recognize that moving from pilot to early expansion and ultimately to population-level scale-up is a challenging and lengthy process.^3^ The ExpandNet/WHO framework is a conceptual tool to guide the analysis of issues to consider in the development of a scale-up strategy or management of a scale-up process.^33^ It serves as the basis for the tool entitled *Nine Steps for Developing a Scaling-Up Strategy*,^34^ which assists stakeholders in developing a scale-up strategy and to manage the process. The ExpandNet/WHO framework has been used to analyze the complex processes of scale-up in a variety of programs and contexts.^35^^–^^37^ It addresses 5 main elements of scaling up: the innovation, environment, user organization, resource team, and 5 strategic choice areas for managing scale-up (Figure 2).^34^

This article uses the ExpandNet/WHO framework to retrospectively analyze the early phases of scale-up of the community distribution of misoprostol to prevent postpartum hemorrhage in Mozambique. The aim of this article is to present a case study that will inform MNCH stakeholders about the barriers and facilitators in the early expansion of the misoprostol program and offer recommendations to stakeholders, both within Mozambique and internationally, of the key components to bring programs to scale.

## METHODS

We used a mixed-methods approach to assess the implementation of the early expansion phase of scaling up misoprostol in 2 provinces in Mozambique. The objectives of this study were to: (1) identify facilitators and barriers to the early expansion of the misoprostol program for the prevention of PPH at the community level, and (2) examine coverage and use of misoprostol in the 2 provinces.

The qualitative component of the study applied a phenomenological approach to understand the experiences of those involved in Mozambique's misoprostol program. Phenomenology is an interpretive approach based on the lived experiences of people who participated in the phenomenon.^38^ Data were collected between February and October 2017 in Maputo City and in 2 districts in Inhambane province and 3 districts in Nampula province. These provinces were chosen due to geographic region—Inhambane is located in the southern region, and Nampula in the northern region of the country. Inhambane province initiated implementation in 2015, whereas Nampula, one of the provinces selected during the second phase of expansion, commenced implementation in 2016. Districts were chosen based on inclusion in the misoprostol program, geographic accessibility, and discussions with provincial and district health authorities.

One-to-one, semistructured qualitative interviews were conducted with (1) MNCH national, provincial, and district stakeholders with experience working on the misoprostol program; (2) health staff (MNCH nurses and midwives, medical chiefs, hospital directors, pharmacists, and health technicians); and (3) CHWs (referred to as *Agentes Polivalentes Elementares* in Mozambique) and TBAs. In addition, focus group discussions were conducted separately with CHWs and TBAs. The ExpandNet/WHO framework and the document entitled *20 Questions for Developing a Scale-up Case Study*^30^ were used to assist in the design of the interview guides for stakeholder and health staff interviews. Focus group discussion questions for CHWs and TBAs focused on the use and understanding of the medication and barriers and facilitators to the misoprostol program. The interview and focus group discussion guides were revised with input from the MOH and local research assistants to ensure questions could be understood in the local language and were relevant to the context.

Participants were recruited via purposive sampling based on advice from key stakeholders in the program and assistance from district health staff and CHWs. We applied the phenomenological approach and interviewed a relatively varied sample of participants engaged in various aspects of the program. We sought to gain a range of experiences rather than selecting an established number of participants. We contacted 18 MNCH stakeholders via email or phone to arrange interviews. Interviews with 19 health staff were organized with assistance from district health authorities who called the health facility in advance. Fifteen of the stakeholders and all of the health staff contacted agreed to be interviewed.

CHWs and TBAs were selected with assistance from the district MNCH coordinator who contacted the CHWs and asked them to come to the health facility for the interview. Where possible, the research team would drive to meet the CHW or TBA at their home or in the community to conduct the interview. In total, we interviewed 15 CHWs and coordinators and 15 TBAs. Three CHWs and 4 TBAs who were contacted were not available to attend an interview due to prior commitments. Additionally, we conducted 4 FGDs with TBAs in Nampula province and 1 FGD with CHWs in each province. Interviews were conducted in English, Portuguese, and local languages where appropriate. The first and second authors conducted the stakeholder interviews. One international and 3 local research assistants trained in qualitative data collection methods and ethical protocols conducted interviews and focus group discussions at the health facility and in the field. Interviews were recorded with permission, transcribed and translated verbatim into Portuguese, and then translated to English. Participant numbers were determined based on obtaining thematic saturation.

National policy and planning documents were analyzed alongside notes and observations from a 2017 national MOH MNCH workshop, which included a review of the misoprostol program. Notes, policy documents, and qualitative interviews were coded and analyzed using NVivo 11 software.

Quantitative data were provided by the provincial health directors at a national MNCH workshop. These data were analyzed to estimate coverage of and access to misoprostol in Inhambane and Nampula provinces from January through September 2017. We present descriptive statistics in the results. The resulting indicators are not based on directly reported data, but primarily based on calculated, indirect data estimates leading to some imprecision.

We organized the data according to the ExpandNet/WHO framework's planning and management categories. Categories of the planning phase included: the environment, the innovation, the user organization, and the resource team. Management of scale-up was coded into the 5 strategic choice areas of the ExpandNet/WHO framework in the following categories: type of scale-up; dissemination and advocacy; organizational process; costs/mobilization of resources; and monitoring and evaluation. We also referenced the *20 Questions for Developing a Scale-Up Case Study*^30^ in coding the transcripts into the planning and management categories. Results were further categorized as facilitators and barriers to scale up, or both. Finally, we included an additional category to report outcomes, including coverage and uptake, to assess progress of the expansion phase. Outcomes were coded into access, utilization, and logistics systems. We defined access as the number of women who delivered with a TBA and received the drug or who received it in advance during an antenatal care visit, as a proportion of the estimated number of expected home births in the catchment area. Utilization was defined as the number of women who used misoprostol (i.e., unreturned doses of misoprostol) as a proportion of the estimated number of expected home births in the catchment area.

Ethical clearance was obtained from the Human Research Ethics Committee at Charles Darwin University, Australia (HREC 2015–2445), the Mozambican National Bioethics Committee, and the MOH. All participants provided informed consent and none requested to be withdrawn from the study.

## RESULTS

In total, we included in the analysis qualitative interviews with 15 stakeholders, 19 health staff, 15 CHWs and coordinators, and 15 TBAs; 6 focus group discussions; and a review of national policy and planning documents.

### Planning the Scale-Up Strategy 2011–2014

#### The Innovation

In the ExpandNet/WHO framework, the innovation refers to “the health service interventions or other new practices that are being scaled up.”^33^ Here, the innovation was the distribution of 600 micrograms of oral misoprostol to pregnant women through 2 channels: (1) during antenatal care visits at 28 weeks or greater to women who meet the criteria for self-administration (Box 2), or (2) via TBAs who administer it to women directly after they give birth at home. The aim of the innovation was to increase access to misoprostol for women who give birth in the community to reduce maternal mortality associated with PPH.

BOX 2 **Eligibility Criteria for Misoprostol Distribution to Pregnant Women for the Prevention of Postpartum Hemorrhage**

A pregnant woman must:

1. Be registered for antenatal care (i.e., have a prenatal health record on file where misoprostol will be registered)
2. Have reached 28 weeks gestation

She must additionally meet at least 1 of these criteria:

1. Have a history of giving birth at home/outside of the health facility
2. Live more than 8 km from a health facility
3. Be a grand multipara (more than 5 previous births)
4. Have a current or past history of multiple pregnancies
5. Have a history of postpartum hemorrhage

Women received misoprostol either directly during an antenatal care visit or through TBAs who administered it to women after they gave birth at home.

**FIGURE 3:**
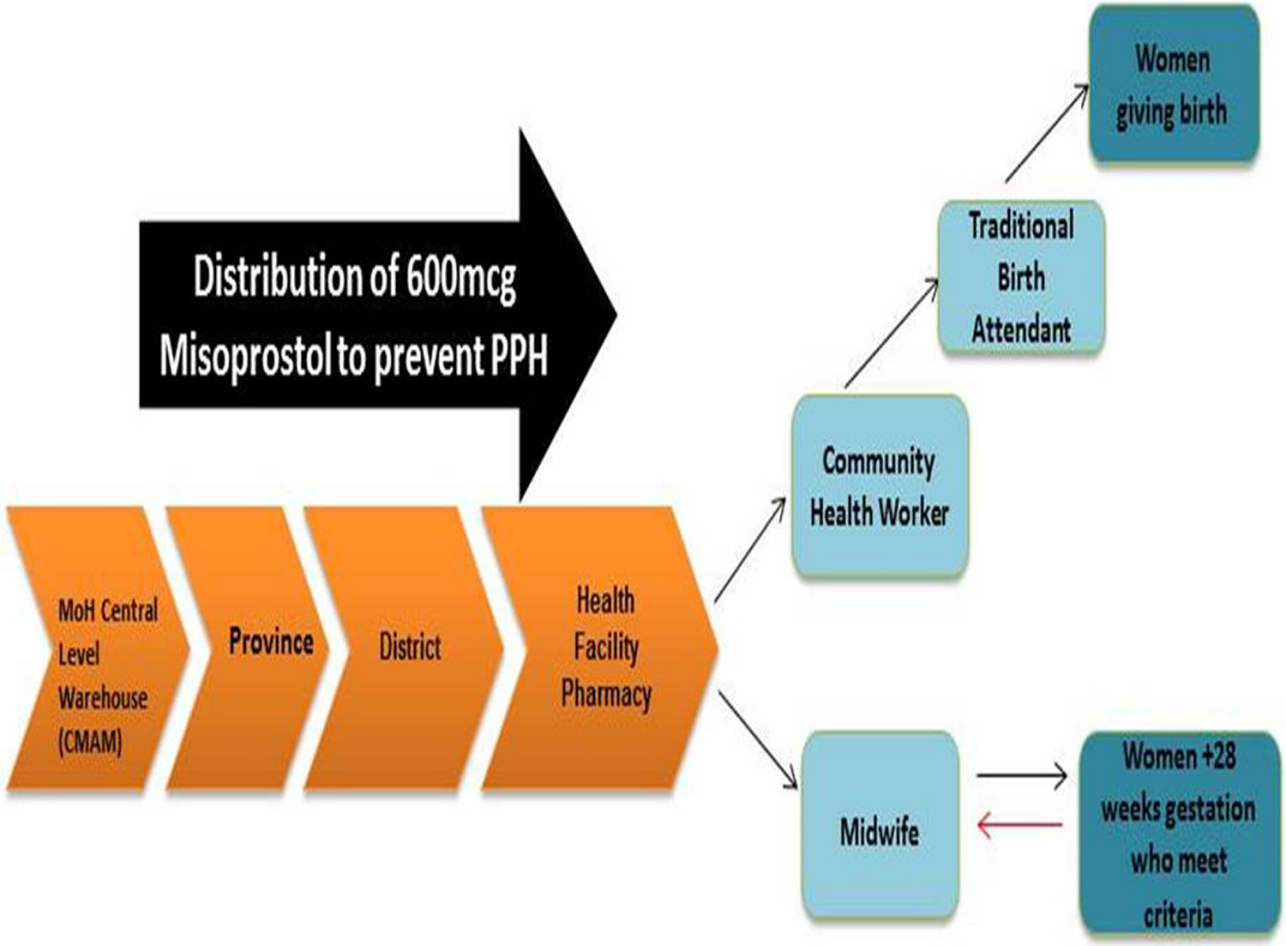
The Innovation: Misoprostol Distribution Chain

A woman who meets the eligibility criteria at her antenatal care visit receives information from the nurse regarding how and when to take misoprostol and a dose (3 pills in a blister pack) to safeguard until her labor. Distribution at the community level occurs between the TBA and the pregnant woman directly after the birth of the baby and before the placenta has been delivered. A CHW provides the TBA with the misoprostol. CHWs, who are salaried employees and have access to a bicycle, are the link between the health facility and TBAs. Upon request, the CHWs usually receive 3 doses (9 pills) of misoprostol, which they then distribute to the TBA(s) in their coverage area monthly (Figure 3).

Some challenges arose with the flow of distribution. In many cases, CHWs lived further from the facility than the selected TBAs. CHWs provided TBAs with the supply of misoprostol, but the CHWs themselves were not trained or permitted to distribute it to pregnant women. In some cases, CHWs lived too far from the TBAs, and they both suggested that the TBAs collect the misoprostol directly from the health facility during monthly TBA meetings. A health staff member from Inhambane province identified this as a major challenge, saying:

*… the only constraint that we have had with the TBAs … is the fact that we do not give misol to them directly.*

CHWs provided TBAs with the supply of misoprostol, but the CHWs themselves were not trained or permitted to distribute it to pregnant women.

This may have limited distribution of misoprostol and reach to remote areas.

During the initial pilot phase, the MOH shifted from a universal distribution strategy, as outlined in the National PPH Strategy, to application of eligibility criteria (Box 2). This shift was proposed by the MOH in response to the number of returned misoprostol doses by women who gave birth at a health facility and to increase controls on the amount of misoprostol in the community. The MNCH Sector Wide Approach (SWAp) Technical Working Group agreed to the implementation of the eligibility criteria in May 2015. The criteria were adopted to target distribution to women deemed most likely to have home births, limit unnecessary distribution, and improve controls on the availability of misoprostol in the community.

This shift impacted the amount of misoprostol required for the program; only 8% of the forecasted stock for universal distribution was needed. The criterion of distance from a facility was explicit (live >8 km from a health facility); however, interpretations of “far from the facility” varied widely among health staff. Some nurses and midwives felt that if a woman lived 10 or 15 km away they would not need misoprostol, as they “should be able to reach the facility” prior to or while in labor. Others defined “far” as 20–30 km from the health facility and thought that only then should women receive the medication. Some MNCH stakeholders and health staff based in the field applauded the additional controls, stating that misoprostol was ‘flooding’ the communities, which increased the risk for potentially using misoprostol for abortion. They also believed that too many doses were returned to the health facility, which produced issues for recordkeeping and disposal. Others criticized the criteria for limiting the number of women who received uterotonic coverage:

*So if we want to prevent PPH it has to be covered universally, it cannot be how it is being done. … this strategy has to be reviewed as soon as possible, it must be universal distribution of misoprostol.* (Stakeholder, Maputo)

A number of stakeholders and health staff in the MOH national MNCH workshop in October 2017 proposed removing the restrictions to improve coverage.

#### Environment

The environment consists of political, sociocultural, and economic factors that impact scale-up and can present opportunities or challenges to expansion.^33^ Several key environmental factors affected the planning and management phases of the misoprostol program, namely the country's financial situation, government support for the initiative, changes to the abortion law, limited capacity of the health system, and wavering support for TBAs.

The country's financial situation was an important environmental factor. In 2016, the Mozambican economy experienced a significant disruption with the revelation of US$1.4 billion in undisclosed debt. The International Monetary Fund halted credit to the country and many international donors, including bilateral governments, withdrew funding. Economic instability was cited as a key issue that could impact scalability of the misoprostol program. Fiscal spending became limited across all government institutions. Many health staff described low motivation with the lack of essential consumables, including gloves for births and vaginal examinations.

Financial constraints were tempered by strong government support for the program. The MOH prioritized the prevention of maternal mortality in the *National Health Sector Strategy 2014–2019*.^11^ The distribution of misoprostol for the prevention of PPH at the health facility is the second objective listed in the strategy to help reduce the MMR. International evidence and advocacy efforts from the Association of Mozambican Obstetricians and Gynaecologists (AMOG) helped garner the support needed for the MOH to approve the expansion of the use of misoprostol across selected districts in Mozambique.

Changes to the abortion law in Mozambique may have positively influenced the political and legal settings in which misoprostol for PPH was introduced. The MOH branded misoprostol for the prevention of PPH as ‘misol’ to differentiate it between misoprostol used for induction of labor or abortion.

The MOH branded misoprostol for postpartum hemorrhage prevention as ‘misol’ to differentiate it from misoprostol used for induction of labor or abortion.

Some central and provincial stakeholders were very supportive of the changes to the legislation and the impact that they would have on improving maternal health. For example, a stakeholder from Maputo explained:

*I think that PPH and safe abortion are very distinct matters. … And when we think that as a country we are limiting the access to misol because we don't want to give access to providers, to women, in case they can misuse this misol … I think we are just limiting access to life.*

However, other stakeholders feared that misoprostol could be misdirected for abortion or labor induction and wanted stricter controls:

*We need to control the misoprostol because other people can use it for other purposes like abortions, and they also know about this use for misoprostol and so we think the district should distribute it to ensure the best control.* (Health Staff, Inhambane)

The environment also includes the capacity of the health system to deliver care and services that impact the success of the planned scale-up.^33^ The limited number of qualified health staff in rural Mozambique resulted in health staff citing they had inadequate time to include distribution of misoprostol to their workload. Few additional resources were provided.

Varying support for working alongside TBAs also impacted planning for the scale-up of misoprostol. Historically, involvement of TBAs within the formal health sector has been limited in Mozambique.^39^ An evaluation of TBA training in one province in 1999 found no impact on MNCH outcomes.^40^ By the year 2000, the MOH shifted the focus on increasing skilled birth attendants to achieve Millennium Development Goal (MDG) 5.^39^ This affected some stakeholders' acceptance of the role of TBAs in the misoprostol program, fearing it would detract from facility-based births:

*Some TBAs charge people and promote these births to be held on her account so she can gain something.* (Stakeholder, Maputo)

Others in favor of TBA participation argued that the health system did not have the capacity to provide a skilled birth attendant for every woman due to a lack of sufficient transport and human resources:

*That woman who lives 25–30 km from the health unit, she will go to prenatal consultation until the sixth, seventh month of pregnancy and then she will not go, and where will she have the baby? She'll go to the [traditional] midwife. In my opinion, it is not to say that the traditional midwife should not help women to give birth, my opinion is to enable the traditional midwife to make a clean birth.* (Stakeholder, Nampula)

These political, sociocultural, and economic factors were all essential components that influenced the planning and management of the misoprostol program.

#### The User Organization

The user organization in the ExpandNet/WHO framework is defined as the institution(s) and/or organization(s) that are expected to adopt and implement the innovation at scale.^33^ The user organization in this case study is the Mozambican MOH, specifically the MOH Department of Women's and Child Health, which leads the implementation of the National PPH Strategy and coordinates monitoring and evaluation (M&E) of the program. Initially, AMOG was selected as the intended user organization to support the MOH to implement the misoprostol program due to their experience in the pilot phase. Later, the MOH deemed they were better suited as the sole user organization given their established infrastructure and staff. The national MOH's leadership role greatly facilitated the expansion of the scale-up as they added to the innovation's credibility and acceptability. However, a number of key national MOH staff members changed positions, and advisors seconded to the ministry moved on. This turnover resulted in delays in finalizing the national PPH strategy and program initiation.

Misoprostol is distributed using the routine supply chain for MNCH commodities, with the addition of CHWs as the distributors between the health facility and the TBAs. In Inhambane province, the supply chain experienced weaknesses in distribution between provincial to district warehouses. Misoprostol stock was procured 1 year prior to the official launch of the program and, as a result, the remaining shelf life of the initial supply was shortened by 1 year. (Misoprostol is a somewhat unstable compound that can degrade quickly when exposed to humidity; it is therefore labeled with a shorter shelf life than most other drugs.)

Provincial MNCH coordinators were requested to lead the misoprostol program with support from the provincial CHW coordinator. Program execution varied widely depending on the leadership of the MNCH coordinators at the district level in each of the 35 implementing districts. Some MNCH coordinators stated that they felt strongly that misoprostol was a lifesaving medication and were committed to the misoprostol program. For example:

*I think it will help a lot as we are in the district, yes deliveries outside the maternity are reducing, for sure, but they continue to occur. This [misoprostol program] is a great asset for us to avoid maternal deaths in the community due to postpartum hemorrhage.* (Health Staff, Inhambane)

Other MNCH coordinators were not very supportive and believed it was not “their program” and instead allocated responsibility to the implementing health facilities.

MNCH nurses were directly responsible for the distribution of misoprostol in advance to pregnant women during antenatal care visits. Some were discouraged by the additional workload associated with the program (e.g., having to ensure the woman met the eligibility criteria, explaining how to use misoprostol and about possible side effects, and recording the information in the register). Several MNCH nurses believed the program was a pilot, especially in Inhambane province where they had experienced multiple hiccups in the supply chain. This negatively impacted their motivation to distribute misoprostol during antenatal care visits or monitor the program. Yet quantitative results revealed that over 80% of all misoprostol distributed was distributed to women in advance during antenatal care visits.

CHWs and TBAs were recruited in coordination with the CHW program and the MNCH department. TBAs were recommended either by the health facility or the CHWs. As a result, recruited TBAs often lived close to the health facility. This was also identified as a key factor in the low coverage experienced in some districts:

*To map all TBAs was very difficult for the health facility and even at the district level. So we said to the MNCH nurses, “Please select first the ones that are reporting to you and then identify some that the CHWs recognize …” Those were the 2 inclusion criteria that we used. We didn't use distance because it was very difficult. … I don't know if we are reaching the TBAs that are really far away.* (Stakeholder, Maputo)

#### The Resource Team

In the planning phase, key members of the resource team included the MOH, AMOG, the United States Agency for International Development (USAID), WHO, Jhpiego's Maternal and Child Health Integrated Program (MCHIP), and the United Nations Population Fund (UNFPA). The latter 2 organizations also provided technical support to the program but were not direct implementers. Jhpiego's Maternal and Child Survival Project (MCSP), which followed from MCHIP, provided essential technical support to the program in Nampula and Sofala provinces. AMOG was involved in the development and implementation of the pilot, along with the NGO Venture Strategies Innovation. Using the evidence from the pilot study, AMOG played a critical role in advocating the need to scale up misoprostol.^41^ UNFPA was responsible for the procurement of stock. The resource team worked together to develop the national PPH strategy.

The resource team members also participate in the MNCH SWAp Technical Working Group. This group has made some critical decisions surrounding the misoprostol program, including the adoption of eligibility criteria for distribution of misoprostol during antenatal care visits. The group reportedly met regularly in 2015 and 2016; however, one informant revealed that the SWAp had not met during the first 10 months of 2017 due to changes in central government staff. The resource team's input and updates regarding progress on the misoprostol program was limited during this period.

### Management of the Scale-Up

Management of the scale-up was categorized as: type of scale-up; dissemination and advocacy; organizational process; costs/resource mobilization; and monitoring and evaluation.

#### Type of Scale-Up

The scaling-up strategy refers to the plans and actions taken to establish the innovation in policies and programs.^33^ The ExpandNet/WHO framework describes 4 types of scale-up: spontaneous, diversification, horizontal (expansion or replication), and vertical (or institutional).^33^ The resource team approached the scale-up of misoprostol through horizontal scale-up via stepwise expansion and also to some extent vertical scale-up through national policy and steps toward institutionalization.

Horizontal scale-up or expansion occurs when innovations are replicated in different geographic locations or are extended to serve larger or new populations. The innovation should be adapted to the new context in the additional location(s). Vertical scale-up occurs when governments reach a formal agreement to adopt the innovation nationally or sub-nationally, the adoption is institutionalized through national planning, policy, or legal processes, and it is implemented nationally or sub-nationally, including maintaining provisions to ensure ongoing effective delivery. Dimensions from both horizontal and vertical scale-up need to be incorporated for an innovation to be sustainable.^33^

The National PPH Strategy outlines a horizontal scale-up strategy, or the expansion of the innovation across different geographical locations to reach a larger population^33^; the expansion of misoprostol focused on 35 districts in the 10 provinces of Mozambique. Districts were chosen based on the following criteria: high incidence of home births; reasonable access to health facilities for women who have obstetric complications; existence of maternal waiting homes; population density; and the existence of CHWs and TBAs who work with the health facility.^29^ In April 2015, the program was initiated in 6 districts in Sofala and Inhambane provinces. The rationale was to allow adequate time for feedback and evaluation before expanding to the remaining 29 districts. District-level stakeholders and health staff identified a number of drawbacks to this strategy, namely, that implementing in 5 districts per province contributed to confusion about whether this was a pilot project and to a lack of trained health staff, as staff are mobile and regularly rotate between implementing and non-implementing districts.

Mozambique's national PPH strategy outlines a horizontal scale-up strategy, focusing first on 35 districts in 10 provinces.

In June 2016, results from Sofala and Inhambane were presented at the MNCH SWAp Technical Working Group meeting in Maputo. Progress in Sofala was limited to 1 district due to conflict that impacted both implementation and M&E. In September 2016, the MOH prioritized trainings to take place in the remaining 29 districts. By June 2017, all 35 districts had commenced distribution of misoprostol (Figure 2).

Vertical scale-up began simultaneously to horizontal expansion via the development of the National PPH Strategy and the beginning of institutionalization into MOH systems, albeit in selected districts. The National PPH Strategy outlined the commitment to incorporating the program into routine MOH systems^29^ (p. 19):

*As PPH is a major cause of maternal death, prevention interventions are part of the larger Ministry of Health plan to reduce maternal mortality involving several National Directorates, Departments and Partners. Thus, during the implementation of the first phase of this strategy, every effort will be made to ensure that interventions and their resource needs are integrated into existing management, funding, coordination and service delivery mechanisms.*

The misoprostol program has yet to be institutionalized. In the early expansion phase, training was delivered to selected MOH staff in the selected districts; UNFPA procured medication and distributed it through the routine MOH supply chain; and M&E was developed parallel to the national health information system.

#### Dissemination and Advocacy

Dissemination involves methods to promote, communicate, and encourage uptake of the innovation by the user organization.^33^ In Mozambique, this included the dissemination of the National PPH Strategy, communication to provincial health staff, and cascade training of health staff and CHWs.

The National PPH Strategy was disseminated from the central to provincial level. The provincial health authorities were responsible for distributing the National PPH Strategy and informing the districts about the program. Memos about the program, particularly around stock, were sent from the central MOH to the provincial health authorities. Communication about the national PPH strategy was a challenge from the beginning, particularly between provincial and district levels but also from the district health offices to the health facilities. Weak flow of information was attributed to insufficient time to disseminate the strategy and induct health staff.

In April 2015, the MOH began training MNCH nurses, CHWs, and TBAs on the distribution of misoprostol for PPH prevention and safe delivery methods. The initial trainings took place in 3 districts in Inhambane and 3 districts in Sofala provinces in 2015. Cadres in Nampula province underwent training for 3 days in August 2016, and distribution of misoprostol commenced in 5 districts in September 2016. In total, the resource team trained 36 master trainers, 548 provincial trainers, and 1,050 CHW supervisors at the health facility level.^42^ Four of the MNCH nurses interviewed were not formally trained in the program as they were not working in the district during the training phase.

In Inhambane significantly more CHWs than TBAs were trained (337 CHWs vs. 47 TBAs), whereas in Nampula a significant number of TBAs were trained (189 CHWs and 980 TBAs). Staff mobility meant that some MNCH staff were not aware of the program or their role. For example:

*We trained the traditional midwives and a nurse from each health unit. We know that there is always movement of the nurses, some nurses do not even know what happened to the misol … if we want to have good results we need to involve all members of the maternal health team, regardless of being in the maternity or prenatal consultation.* (Health Staff, Nampula)

While nurses and midwives were generally supportive of the program some hesitated to distribute misoprostol without formal training.

National-level advocacy played an important role in the decision to scale-up the innovation due to divisions among stakeholders about initiating the misoprostol program. The main hesitations were fears that misoprostol would potentially detract from facility-based births and/or would be used for abortion. The MNCH SWAp group reviewed global studies and the MOH requested that a pilot be conducted in Mozambique to assess whether misoprostol could be distributed safely during antenatal care visits and by TBAs.^24^ This review, alongside the results of the Mozambican pilot and meetings with AMOG and other MNCH stakeholders about the benefits of misoprostol, eased fears about controlling the medication in the community. After discussions, the MOH agreed to allow the distribution of misoprostol for the prevention of PPH at the community level.

#### Organizational Process

Organizing the process of scale-up involves those responsible for decision making and implementation.^33^ In the case of Mozambique, the central MOH was the critical decision maker and communication about the misoprostol program came from the central to the provincial health authorities. The provincial health authorities were then expected to implement the program in partnership with the district health authorities and health staff. Central-level government involvement was a key facilitator for the initial phase of introducing misoprostol into some parts of the health system. However, coordination and communication between the levels was a barrier. In speaking about the barriers of managing the system, a stakeholder from Maputo revealed:

*When you had misol at the provincial warehouse then it was difficult sometimes to get the misol to the district warehouse. So we had this challenge at the time - people, values, managing the system; all this was challenging to improve the system for misol to flow.*

The organizational process was also marked by gradual scale-up starting with only 6 districts in 1 year. Given the delays initiating the program, rapid rollout in the remaining 29 districts occurred over just 6 months in response to pressure from the national MOH to implement in all of the selected 35 districts. This limited time for monitoring and lessons learned to be considered.

#### Costs/Resource Mobilization

A budget of US$2.6 million was outlined in the National PPH Strategy for the first 2 years of the program.^29^ The MOH budget covered the fixed operational and human resources costs for ongoing delivery of services. Short external donor grants were the main source of upfront program funding. Partners within the resource team provided some financial resources to initiate the rollout. Originally, USAID planned to provide both technical support and funding to procure misoprostol. However, they raised concerns surrounding the supervision and controls of the medication at the community level and requested further measures to ensure that it would not be misdirected and used for abortion in the community. This led to several months of delays. UNFPA then agreed to procure misoprostol and became the major donor, funding both the procurement and training components. Other partners, including Jhpiego, provided funding for training in 2 provinces where they supported MNCH projects.

In 2015, Jhpiego's MSCP project also provided a 1-year grant to AMOG to provide technical support including supervision. In early 2017, the initial grant from UNFPA expired and the program continued on a limited budget. UNFPA received funding from the UK Department for International Development (DFID) for 2018–2020 to continue support for the program. However, limited funds were allocated to the program outside of those for procurement, training, and the production of communication materials. The lack of available funding was cited as a major barrier to ensuring the program's sustainability, ongoing supervision, and evaluation.

#### Monitoring and Evaluation

The misoprostol M&E system was developed in parallel to both the national MNCH and CHW databases and separate to the national health information system. Data collection on misoprostol indicators represented additional tasks for the district MNCH nurses, who, without a clear system, were documenting returned doses of misoprostol by writing data on a separate paper or creating new columns in the prenatal registers. As a result, health staff at every level were concerned about the validity of monthly data collation:

*Sometimes we send a message, ‘Colleague tell me there, when and how many pills you distributed and to the CHW too,’ she [MNCH nurse] will give you that information. Then you add it and you feel that there is some number missing but you cannot confirm why because you do not have a concrete instrument for evaluating the information.* (Stakeholder, Provincial Level)

The misoprostol M&E system was developed in parallel to the national health information system.

Furthermore, no additional funding was provided at the provincial or district level to undertake supervision visits. Between 2015 and 2017, the MNCH staff alongside partners, including UNFPA, Jhpiego, and AMOG, conducted several isolated supervision visits, the findings of which were not widely disseminated. Some provincial MNCH coordinators were able to monitor alongside supervisory visits to other programs, but overall they felt that time spent was insufficient. Similarly, some district MNCH coordinators felt that they did not have the budget, time, or mandate to supervise the program. In other cases, district MNCH nurses were very involved in the program. Many stakeholders in Maputo and at the provincial level felt that the lack of M&E was a major barrier for the sustainability of the program.

### Outcomes

#### Access and Utilization

Program data, collated by the provincial MNCH coordinators for the MOH MNCH workshop held in Maputo in October 2017, were used to estimate access to and utilization of misoprostol for the first 9 months of 2017 (Table 1). Weaknesses in the M&E system may have resulted in discrepancies in both numerators and denominators.

**TABLE 1.** Estimated Access to and Utilization of Misoprostol to Prevent Postpartum Hemorrhage in Inhambane and Nampula Provinces, Mozambique, January–September 2017

Location	A	B	C	D	E	F	G	H	I	J	K
	Misoprostol Distributed Through ANC and TBAs	Misoprostol Distributed Minus Returned	District Population (2017)	Annual Births in District (2017)^a^	Proportion of Health Facilities Enrolled in Misoprostol Program	Annual Births in Misoprostol Areas^b^	Births in Misoprostol Areas (First 9 Months of 2017)^c^	Home Births in Misoprostol Areas (First 9 Months of 2017)^d^	Access to Misoprostol at Home Births^e^	Utilization of Misoprostol at Home Births^f^	Interpretation
**Inhambane Province**											
Zavala	616	243	163,620	7,363	38%	2,798	2,098	630	98%	39%	Excellent access, poor utilization
Homoine	417	45	131,680	5,926	46%	2,726	2,044	613	68%	7%	Fair access, very poor utilization
Total both districts	1,033	288	295,300	13,289	42%	5,581	4,143	1243	83%	23%	Good access, poor utilization
**Nampula Province**											
Mecuburi	1,464	840	189,880	8,545	43%	3,674	2,756	827	100%^g^	100%^g^	Excellent access and utilization
Erati	2,568	2,034	322,737	14,523	54%	7,842	5,882	1,765	100%^g^	100%^g^	Excellent access and utilization
Monapo	1,357	1,039	389,902	17,546	43%	7,545	5,659	1,698	80%	61%	Good access, fair utilization
Total 3 districts	5,389	3,913	902,519	40,613	47%	19,088	14,296	4,289	100%^g^	91%	Excellent access and utilization

a Column C * 4.5%.

b Column E * Column D. The calculation uses a fixed number of residents and assumes population figures are similar across the country, leading to imprecision in the calculation of access and utilization indicators.

c Column D * 0.75.

d Column G * 0.30.

e Column A/Column H.

f Column B/Column H.

g In Mecuburi, access was 100% with 637 remaining doses and 13 additional doses utilized; Erati had 100% access with 803 remaining doses and 269 additional doses utilized. In total, there were 1,440 remaining doses in all 3 districts of Nampula.

There was large variation in overall access and utilization between districts and the 2 provinces. Access to misoprostol in Nampula exceeded Inhambane province by 17%, and utilization was much greater in Nampula (91%) than in Inhambane (23%). Two districts in Nampula reported over 100% coverage of misoprostol of women who gave birth at home. Coverage is a combined measure of both access and utilization of misoprostol (Table 1).

Access to and utilization of misoprostol varied widely between districts and between the 2 provinces.

In Inhambane, we found a number of possible reasons for low coverage. Health staff restricted access to misoprostol justified by using the eligibility criteria. There were also frequent stock-outs. TBAs and health staff reported that their priority was to ensure women gave birth at the health facility. Therefore, TBAs said they administered misoprostol only to women who had “surprise” births at home or who had “birthed along the way” to the health facility, as they indicated they always encouraged women to give birth at a health facility.

In Nampula, higher coverage than Inhambane may be in part attributable to the institutional memory of the 2009–2010 pilot study: health staff and CHWs had previous training and were familiar with and supportive of the program. Distribution in Nampula started almost 1 year after Inhambane, such that we may have captured a period of initial high motivation. Political champions for misoprostol in Nampula province may also have positively impacted MNCH health staff attitudes and willingness to implement the program. There were significantly more TBAs trained and involved in the program than Inhambane (980 vs. 47, respectively). Further, the program in Nampula benefited from technical support from Jhpiego's MSCP program. See Table 2 for a comparison of inputs.

**TABLE 2.** Program Inputs Provided in Inhamabane vs. Nampula Provinces, Mozambique

Inputs	Inhambane	Nampula
Supervision	Ad hoc supervision from health staff when time/resources permitted	Routine supervision from Jhpiego MCSP staff Some initial supervision visits from AMOG (funded by MCSP)
Personnel	Health staff strongly believed the misoprostol program was a pilot project as it was only in selected districts in the province Strictly implemented eligibility criteria Significantly less misoprostol distributed at ANC (989 doses) than Nampula Fear of misuse limited distribution	Greater sense of support from health staff as many were aware of the 2009–2010 pilot and appreciated the potential misoprostol has to reduce PPH and MMR Less sense of a need to limit women due to criteria Significantly more distributed at ANC (13,602 doses) than Inhambane
Champions	Lack of clear champion; MNCH leaders supportive yet constrained by lack of resources	Provincial and district MNCH leaders showed very strong support for the program and encouraged implementation
Training	Funded by UNFPA; led by trained MOH master trainers, with UNFPA technical support Training imbalanced; targeted more CHWs (337) than TBAs (47)	Funded by Jhpiego's MCSP program; led by AMOG and MOH with MCSP technical support Provided significantly more TBAs with training (980), providing greater community coverage
Logistics	Challenges distributing stock from province to districts; as of October 2017, 87% of misoprostol stock remained in provincial warehouse	Fewer challenges distributing stock from province to districts; only 1% of stock remained in provincial warehouse as of October 2017
Monitoring and evaluation	Parallel system; not integrated in the national health information system	Parallel system; not integrated in the national health information system MCSP provided technical support to develop M&E tools but they were not adopted at the national level No data available provincially on misoprostol returns from CHWs/TBAs

Abbreviations: AMOG, Association of Mozambican Obstetricians and Gynaecologists; ANC, antenatal care; CHW, community health worker; MCSP, Maternal and Child Survival Project; M&E, monitoring and evaluation; MMR, maternal mortality ratio; MNCH, maternal, newborn, and child health; MOH, Ministry of Health; PPH, postpartum haemorrhage; TBA, traditional birth attendant; UNFPA, United Nations Population Fund.

Table 3 reveals that from January through October 2017, MNCH nurses in Nampula distributed significantly more misoprostol during antenatal care visits than in Inhambane (13,602 doses vs. 989, respectively). In both provinces, the majority of misoprostol was distributed during antenatal care visits rather than by TBAs. In Inhambane, 194 doses provided to the CHWs were not accounted for (not provided to the TBA nor returned to the antenatal care clinic). No data were available provincially for misoprostol returns from CHWs or TBAs in Nampula, although we found individual health centers recording returns.

The majority of misoprostol was distributed during antenatal care visits rather than by TBAs.

**TABLE 3.** Distributed and Returned Misoprostol by Cadre, January–October 2017

Province	Total Distributed at ANC	Total Distributed by CHWs	Total Distributed by TBAs	% of Total Distributed to CHWs Reaching TBAs^a^	% of Total Distributed at ANC via TBAs^b^	Returned to ANC	Returned to ANC by CHW or TBA
Inhambane (Homoine, Zavala districts)	989	325	44	14%	4.4%	201	87
Nampula (Mecurburi, Erati, Monapo districts)	13,602	5,578	900	16%	6.6%	1662	Unknown

Abbreviations: ANC, antenatal care; CHW, community health worker; TBA, traditional birth attendant.

a Total distributed by TBAs divided by total distributed by CHWs * 100.

b Total distributed by TBAs divided by total distributed at ANC * 100.

#### Logistics System

The initial quantity of misoprostol distributed from the central to the provincial level was based on the projection for 100% coverage of home deliveries in the chosen districts. The introduction of eligibility criteria in 2015 reduced the number of eligible women. This led to an excess of misoprostol available at all levels. Much of this stock was never distributed, resulting in the expiry and subsequent incineration of the first wave of commodities.

As of October 2017, in Inhambane 87% of misoprostol stock was available at the provincial warehouse, but only 13% had been distributed to the district or health facility level. In contrast, in Nampula, almost all of the stock (99%) was distributed to the district health facilities. Informants in Inhambane stated that this was due in part to lack of leadership from some district medical chiefs to request adequate misoprostol stock, as well as hesitation by pharmacists to fill requisitions. Further inquiry suggested that pharmacists and some medical chiefs were hesitant to distribute what was previously a medication with the highest restrictions into the community and feared it would be used for abortion. One health staff said the medication was kept “locked under 7 doors” due to concerns about the controls in the community and the maternity units. Some informants felt that this was one of the largest barriers to reaching high coverage. Notably, pharmacists were not included in the dissemination of the National PPH Strategy.

Lack of availability of the commodity at district level was a key reason for low coverage in Inhambane. From September 2016 through June 2017, misoprostol stock was rationed in Inhambane with only 5–10 doses distributed per participating health facility. One district pharmacist reported requesting 1,000 doses and received only 30 doses. Records revealed that other districts requested very small amounts—30 or 60 pills at a time (sufficient for 10–20 women).

At the time of publication, the MOH had not yet set a timeline to achieve national scale-up. Between 2018 and 2020, the MOH will continue to focus on strengthening the program in the existing 35 districts and improving M&E mechanisms before further expansion.

## DISCUSSION

Our analysis of factors that facilitated scale up of use of misoprostol to prevent postpartum hemorrhage (Table 4) suggest that the political environment was critical in allowing adoption of the innovation, benefiting from a high level of national political support, particularly as reduction of maternal mortality is the first indicator in the *Mozambique Health Sector Strategic Plan 2014–2019*. The misoprostol program was central to the National PPH Strategy and facilitated by national leadership, allowing for inclusion into some of the MOH infrastructure, systems, and human resources.

**TABLE 4.** Facilitators and Barriers to Scaling Up Misoprostol for the Prevention of Postpartum Hemorrhage in Mozambique, by ExpandNet/WHO Framework

Factors	Facilitator	Barrier
***Planning Phase***		
**Environment**		
1. Financial situation		✓
2. Government support including champions	✓	
3. Changes in abortion law	✓	
4. Limited capacity of health system		✓
5. Wavering support for TBAs		✓
**Innovation**		
1. Clear, concise, well-defined	✓	
2. Adaptation of criteria		✓
3. Flow of distribution		✓
**User Organization**		
1. MOH Central	✓	
2. MOH MNCH health staff	✓	✓
3. MOH pharmacists		✓
4. APE (dependent on TBA relationship and distance)	✓	✓
5. TBA recruitment (close to health facility)		✓
**Resource Team**		
1. Members	✓	✓
2. Existence of SWAp MNCH Technical Working Group	✓	
3. SWAp MNCH Technical Working Group irregularity of meetings		✓
**Management Phase**		
**Type of Scale-Up**		
1. Horizontal (phased expansion)	✓	
2. Limited sites in each district (5 health facilities in selected districts)		✓
3. Untrained health staff due to mobility		✓
4. Vertical (institutionalization)	✓	
**Dissemination and Advocacy**		✓
1. Development of National PPH Strategy	✓	
2. Communication of PPH Strategy		✓
3. Training of health staff, APEs, and TBAs	✓	✓
**Organizational Process**		
1. MOH Central	✓	
2. MOH Provincial	✓	✓
3. MOH District		✓
**Costs/Resource Mobilization**		
1. Available Budget		✓
**Outcomes**		
1. Utilization and access in Nampula province	✓	
2. Utilization and access in Inhambane province		✓
3. Logistics system		✓

Abbreviations: APE, Agentes Polivalentes Elementares (community health worker); MNCH, maternal, newborn, and child health; MOH, Ministry of Health; PPH, postpartum hemorrhage; SWAp, Sector Wide Approach; TBA, traditional birth attendant; WHO, World Health Organization.

While the MOH led the program planning and implementation process, misoprostol is being used for the prevention of postpartum hemorrhage only within selected districts and has yet to be incorporated into routine MOH systems. The inputs outlined in Table 2 suggest that Jhpiego's MCSP program may have been a factor in Nampula's success at achieving greater coverage, particularly surrounding supervision and dissemination of information via communication materials. Institutionalization into the existing government priorities and health system is a critical factor to ensure the program is supported nationally.^43^ Until misoprostol is fully integrated into the national health system and long-term funding is secured, it may continue to be viewed as a vertical project.

AMOG and partners employed a successful advocacy strategy to encourage the MOH and MNCH SWAp members of the potential high impact misoprostol had on improving incidence of PPH. The innovation was equally supported by international evidence and positive results from the local pilot study.

The program benefited from the strong existing network of CHWs and TBAs who provided the local capacity for implementation and credibility. However, very few TBAs received misoprostol for direct distribution (Table 3) compared with the supply that was provided to the CHWs. This reveals that there was a weakness in distribution between CHWs and TBAs in both provinces, which coincides with qualitative interviews that found some TBAs were having challenges receiving misoprostol from CHWs. Furthermore, in Inhambane significantly more CHWs were trained versus TBAs, whereas the opposite occurred in Nampula. This may have been due to the number of TBAs available and willing to participate in the misoprostol program.

Weaknesses in distribution of misoprostol between CHWs and TBAs existed in both provinces.

We provide a number of recommendations for the MOH and resource team based on the lessons learned in the expansion phase (Box 3). There is clearly a need to allow flexibility in who can distribute misoprostol. In areas that do not have TBAs, CHWs could potentially distribute misoprostol in advance to women in addition to advance distribution at antenatal care visits. A number of studies in Ethiopia, Ghana, Nepal, Nigeria, and Rwanda have shown that CHWs can correctly and effectively distribute misoprostol to women.^44^^–^^46^ TBAs emphasized their role in supporting women to give birth at the health facility. While positive, this may also have implications for detracting from safe delivery for women who are encouraged by TBAs to walk for kilometers in labor to attempt to reach the health facility and instead give birth on the side of the road, in transit. This finding was reported and discussed in more detail in a related article.^47^

BOX 3 **Key Recommendations to the Mozambique Ministry of Health and Resource Team**

**The Innovation**

- Distribute misoprostol directly to TBAs in situations where there are no CHWs
- In areas without TBAs, allow CHWs to distribute misoprostol in advance to pregnant women in their catchment area
- Adjust one or more criteria:
  - Reduce the timeline to receive misoprostol at ANC from 28 weeks gestation to 24 weeks
  - Reduce the distance to 5 km or measure it in time (e.g., 30 minutes or 1 hour walking)

**Dissemination and Advocacy**

- Widely disseminate the National PPH Strategy to all health staff including pharmacists and provincial and district authorities
- Implement a human rights-based approach that advocates to health staff and communities that women have a right to access misoprostol
- Increase demand in the community by disseminating information about the benefits of misoprostol, how to safely use it, and where to access it via CHWs, TBAs, and mobile health units

**Organizational Process**

- Undertake a formal review of the misoprostol program to identify needed adaptations and develop a systematic scale-up strategy for the next phases
- Medical chiefs and pharmacists should ensure a consistent supply of misoprostol, which meets the demand; review the push/pull system to allow for timely requisitions of misoprostol
- Train more TBAs and health staff to increase the number of qualified people who can distribute misoprostol

**Costs/Resource Mobilization**

- Develop a long-term plan for resource mobilization or redistribution of resources to fund the misoprostol program and next phase of scale-up

**Monitoring and Evaluation**

- Provide technical support to introduce the misoprostol indicators within the national health information system
- Appoint provincial focal persons to support timely reporting and data analysis
- Provide a formal record/system to accurately record misoprostol provided to CHWS and returns of misoprostol

Overall, the innovation was well-defined, credible, and adapted to the context. However, the introduction of restrictive eligibility criteria further complicated the distribution, limiting which women were targeted. The distance criteria was particularly contested by stakeholders and corroborated by health staff who made arbitrary decisions about who could or could not feasibly walk to the health facility while in labor. Applying eligibility criteria placed emphasis on facility-based birth instead of increasing uterotonic coverage. Due to the difficulty in assessing who is likely to experience PPH, misoprostol is often implemented as a population-based universal prevention method.^46^^,^^48^^–^^50^ We recommend revising the eligibility criteria to increase the number of women targeted to receive misoprostol in advance at antenatal care visits.

Four of the nurses and midwives interviewed had not received training in the misoprostol program, limiting their involvement and impact. Fewer than half of all TBAs have been trained on the use of misoprostol and distance was not considered in recruitment. As a result, many women who give birth at home are not receiving misoprostol via their TBA. Both of these factors may have contributed to reduced coverage. We recommend future expansion plans consider implementation of the misoprostol program within all health facilities in the selected districts and/or province and training all health staff including pharmacists, CHWs, and TBAs to avoid these gaps in program implementation.

The budget for the misoprostol program was not fully funded by the government and depended on external funding. Funding is an essential component of catalyzing and sustaining scale-up, and the nature of short-term donor grants can impede a government's capacity to achieve national scale-up.^31^ While some organizations assisted with the costs of training and provided technical support, the government and partners did not commit sufficient resources for adequate M&E. We recommend the resource team provide funding for supervision as well as technical support to introduce the misoprostol indicators within the national health information system to improve efficiency and remove the existing parallel data collection system. M&E is an element for successful scale-up as outlined in the ExpandNet/WHO framework and other scale-up literature.^31^^,^^51^

The study revealed disparity between the coverage and access of misoprostol for PPH prevention in Inhambane and Nampula provinces. A number of factors may have resulted in inflated numerators. Some CHWs may have maintained their supplies that had yet to be distributed to TBAs or returned to the health facilities. One supervisor found duplication, where both a TBA and her client had received misoprostol. Given our finding that there was no space in the MNCH registers for returned misoprostol (Table 1, Column B), returned pills may not have been recorded. We found no evidence of diversion in this study. Denominators using the estimate of 4.5% of women of reproductive age also likely underestimate the population, given the high fertility rate in misoprostol target areas.

In 2017, Inhambane reported good access (83% of expected users received misoprostol) yet limited utilization (23% of women who received misoprostol used it). Limited utilization may have been due to the emphasis on facility births in the province resulting in women coming to the health facility to give birth, or perhaps due to limited communication to the community surrounding the purpose of misoprostol. The barriers presented in Table 4 may have impacted the outcomes, specifically the strict application of the criteria; retention of trained staff; limited availability of stock at district level; limited participation of TBAs who live in more distant rural areas; and minimal support and resources provided.

On the other hand, high coverage and utilization was found in Nampula province, resulting in high uterotonic coverage at home births. This was perhaps due to the program being in the first year of implementation, provincial MNCH champions who encouraged implementation, a greater number of participating TBAs, and the additional benefits of technical support from an NGO, particularly relating to supervision. The original pilot took place in Nampula, which may have also positively contributed to increased buy-in from local MNCH leaders, health staff, CHWs, and TBAs.

The lack of a data collection tool to record returned misoprostol was a barrier. In Inhambane, approximately 200 doses provided to CHWs were unaccounted and there was no data provided regarding misoprostol returns from CHWs or TBAs in Nampula. This may be attributed to the following: (1) most CHWs kept the medication in their drug kit for months until the TBA requested the misoprostol; (2) recording returns was not a formal process in the antenatal care clinics and not always recorded; (3) some CHWs directly distributed the medication to women where there was no TBA available; or (4) potentially misdirected for abortion or induction; however, there was no evidence to support this.

The program experienced significant difficulties with the logistics system to distribute the misoprostol, which should be anticipated and addressed as part of program planning and M&E. The majority of misoprostol was distributed in advance at antenatal care clinics with less than 20% distributed to CHWs to provide to TBAs (Table 3). The introduction of CHWs as the middle distributor from the health facility to the TBAs added an additional step and increased the complexity of the logistics system. Challenges managing the commodity are common in scale-up programs.^43^^,^^52^ In Mozambique, this phenomenon is not unique to misoprostol. A study in Sofala province found that between 2011 and 2013, 85% of district warehouses (n=13) experienced a stock-out of an essential drug at least once in the 3 annual stock assessments.^53^ Medicine stock-outs were strongly associated with distance from the district warehouse to the health facility. However, in 2015 a UNFPA assessment found that 84% of health facilities across Mozambique had at least 7 essential maternal and reproductive health medicines, marking an improvement from the previous assessment of 59% in 2014.^54^

Concerns that misoprostol for the prevention of PPH would be used by CHWs, TBAs, or women for abortion impacted implementation. First, the program faced initial delays at the central level due to stakeholder concerns about the potential of incorrect use at the community level, and the need for further supervision delayed initial donor support for the misoprostol program in Mozambique.^41^ Second, the MNCH SWAp Technical Working Group introduced eligibility criteria to impose further restrictions and limit distribution at the community level. Third, there was hesitation by some provincial and district medical chiefs and pharmacists to distribute misoprostol to CHWs and TBAs. In Mozambique, opponents of misoprostol for the prevention of PPH often fear that it will be used incorrectly or for abortion.^41^ However, our study found no evidence of confusion about the objective of misoprostol for the prevention of PPH or diversion of the drug for induction of labor or abortion.

Communication, advocacy, and a human rights-based approach that reinforces that women have a right to access this lifesaving medication is needed from health leaders and champions to shift this culture of fear and ensure all staff understand the aims and protocol of the misoprostol program. The delivery gap—when public health professionals know what is effective and yet a gap remains on how the intervention is executed—remains a critical issue to implementation of health innovations.^55^ Due to delays initiating the program in the first 6 districts, roll-out in the remaining 29 districts took place relatively quickly, arguably without adequate time for reflection and lessons learned. We recommend that the resource team and relevant stakeholders identify needed adaptations and develop a systematic strategy to guide future scale-up. We further recommend that the MOH and resource team improve communication and understanding of the National PPH Strategy and commit to incorporating the misoprostol program into the MOH institutional systems, including the national health information system.

### Limitations

We interviewed national-level stakeholders and analyzed national documents and meeting notes. However, fieldwork was based in only 2 of the 10 provinces undertaking the misoprostol program. Inhambane province and Nampula province experienced differences in program inputs, resources, and outcomes. This limits the generalizability of the study to Mozambique and to other countries. This case study cannot project the impact of the program and analysis is limited to the early expansion phase. It is also important to note that this was a retrospective analysis of the planning and management of the misoprostol program using the ExpandNet/WHO framework; the framework was not explicitly utilized by the user group or resource team in Mozambique as a guide in their scale-up efforts. Lack of availability of quantitative data limited analysis and therefore we were only able to provide estimates of the coverage of misoprostol in the first 9 months of 2017. Furthermore, data on access to and utilization of misoprostol were based primarily on calculated, indirect estimates, not directly reported data, leading to imprecision.

## CONCLUSION

This study provided a retrospective analysis of the planning and the management of the early expansion of the scale-up of misoprostol for PPH prevention. The ExpandNet/WHO framework is a useful tool to plan, track progress, and allow for continuous learning. While the framework was not used in the planning or management of the misoprostol program in Mozambique, the scale-up effort would have benefited from the development of a more systematic scale-up strategy during the initial planning phase.

This case study identified barriers and facilitators to scale-up, as well as recommendations for the misoprostol program rooted in the ExpandNet/WHO framework. The Mozambican misoprostol program benefits from political support, inclusion within the National PPH Strategy, and integration into some of the MOH infrastructure, systems, and human resources. Between 2018 and 2020, the MOH and resource team will focus on implementation in the existing 35 districts. This study identifies the need to have a formal review of the misoprostol program with the MOH, resource team, and other stakeholders to identify lessons learned and needed adaptations, and to develop a systematic scale-up strategy to guide the continued national scale-up process.

## Notes

**Peer Reviewed**

**Funding**: PhD Research Training Program, Australian Government.
